# Relative efficacy of three approaches to mitigate Crown-of-Thorns Starfish outbreaks on Australia’s Great Barrier Reef

**DOI:** 10.1038/s41598-020-69466-1

**Published:** 2020-07-28

**Authors:** David A. Westcott, Cameron S. Fletcher, Frederieke J. Kroon, Russell C. Babcock, Eva E. Plagányi, Morgan S. Pratchett, Mary C. Bonin

**Affiliations:** 1 CSIRO, Land and Water, Atherton, QLD 4883 Australia; 2 Australian Institute of Marine Sciences, Townsville, QLD 4810 Australia; 3 CSIRO, Oceans and Atmosphere, P.O. Box 2538, Brisbane, QLD 4072 Australia; 4 ARC Centre of Excellence for Coral Reef Studies, James Cook University, Townsville, QLD 4810 Australia; 5 Great Barrier Reef Marine Park Authority, Townsville, QLD 4883 Australia

**Keywords:** Conservation biology, Tropical ecology, Conservation biology, Ecology, Ecology, Ocean sciences

## Abstract

Population outbreaks of Crown-of-Thorns Starfish (COTS; *Acanthaster* spp.) are a major contributor to loss of hard coral throughout the Indo-Pacific. On Australia’s Great Barrier Reef (GBR), management interventions have evolved over four COTS outbreaks to include: (1) manual COTS control, (2) Marine Protected Area (MPA) zoning, and, (3) water quality improvement. Here we evaluate the contribution of these three approaches to managing population outbreaks of COTS to minimize coral loss. Strategic manual control at sites reduced COTS numbers, including larger, more fecund and damaging individuals. Sustained reduction in COTS densities and improvements in hard coral cover at a site were achieved through repeated control visits. MPAs influenced initial COTS densities but only marginally influenced final hard coral cover following COTS control. Water quality improvement programs have achieved only marginal reductions in river nutrient loads delivered to the GBR and the study region. This, a subsequent COTS outbreak, and declining coral cover across the region suggest their contributions are negligible. These findings support manual control as the most direct, and only effective, means of reducing COTS densities and improving hard coral cover currently available at a site. We provide recommendations for improving control program effectiveness with application to supporting reef resilience across the Indo-Pacific.

## Introduction

Crown-of-Thorns Starfish (COTS; *Acanthaster* spp.) are among the largest and most efficient coral predators, and COTS population irruptions (often termed ‘outbreaks’) are a major contributor to coral loss throughout the Indo-Pacific region^1^. To address increasing and elevated densities of COTS, manual control programs, mainly involving lethal injections in situ^2^ or hand collections and disposal on shore^3^, have been implemented since the 1960s. By 2014 some 84 manual control programs had been conducted across the Indo-Pacific, killing or removing at least 17 million starfish at an estimated cost of US$15–44 million^1^. Such programs have generally had limited success in either suppressing COTS densities or preventing coral loss^3–5^. Several authors have suggested that successful manual control is unlikely with large scale or severe COTS population outbreaks^6–9^. Given that single-injection methods for culling individual COTS are now close to 100% effective^10^, significant improvements in manual control programs are likely to be found in the strategic and effective deployment of in-water resources to manage COTS impacts on coral reefs^11,12^. Despite this, there is little information available on the efficacy of manual COTS control programs nor on their performance relative to other measures implemented to manage population outbreaks of COTS to minimize coral loss.

The Great Barrier Reef Marine Park (GBR) is one of the world’s largest coral reef ecosystems and a globally recognized world heritage area. Despite its iconic status, the GBR is subject to a range of anthropogenic threats, and, like tropical coral reefs around the world^13,14^, it has experienced significant degradation in recent decades^15–18^. During the period 1985–2012, average hard coral cover across the GBR halved, which was in large part attributed to recurrent population outbreaks of the Pacific Crown-of-Thorns Starfish, *Acanthaster* cf*. solaris*^15^. Climate change, and specifically ocean warming has since emerged as the major cause of coral loss and reef degradation on the GBR, causing extensive mass-bleaching and mass-mortality of corals in the far-northern and northern GBR during 2016 and 2017^14^. Simultaneously, however, the GBR has been experiencing renewed outbreaks of COTS which started in 2008–2010 in the northern GBR^1^, and are currently concentrated on reefs in the central and southern GBR. Notably, high densities of adult COTS are now occurring between Townsville and Mackay^19^, causing greatest coral loss in areas that were mostly unaffected by recent mass-bleaching^17^. The persistence of these outbreaks directly undermines reef resilience by adding to coral mortality, suppressing coral recruitment and recovery^20^, and interfering in the capacity of corals to acclimate and adapt to changing environmental conditions.

**Figure 1:**
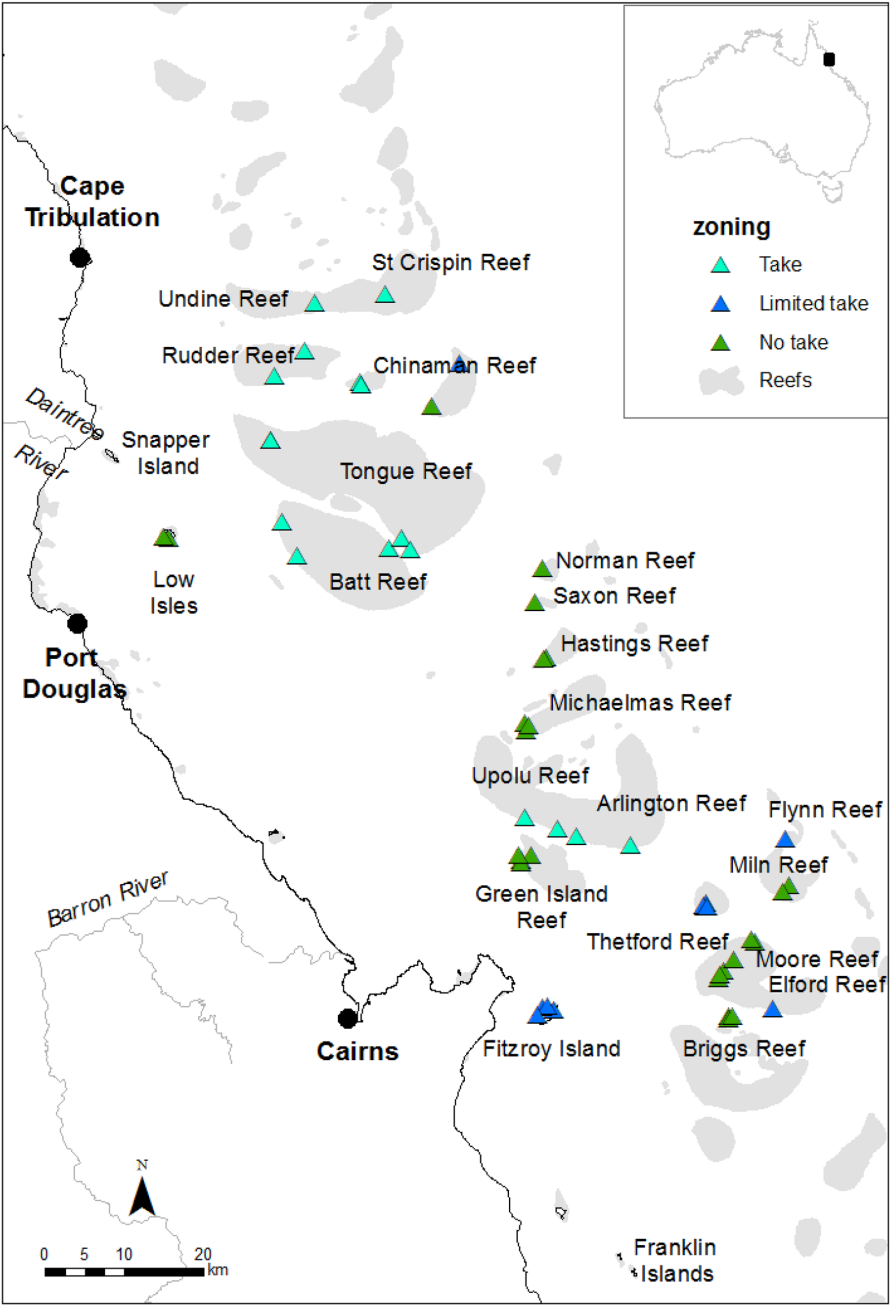
Site locations for manual control of the Pacific Crown-of-Thorns Starfish (COTS), *Acanthaster* cf*. solaris*. Manual control of COTS took place at a total of 52 sites at 21 reefs in three different types of spatial zoning in the Cairns sector of the Great Barrier Reef from July 2013 to December 2017 (light blue = take zones, dark blue = limited take, green = no take). Data on COTS abundance from the COTS control program, and coral cover from the Great Barrier Reef Marine Park Authority’s Reef Health Impact Surveys, were collected at each of the 52 sites during this period. Insert shows location of study area in Australia. The spatial layers to create the map were obtained from the Great Barrier Reef Marine Park Authority under a Creative Commons Attribution 4.0 licence (CC BY) (https://www.gbrmpa.gov.au/about-us/resources-and-publications/spatial-data-information-services).

Sustained and ongoing degradation of coral reefs globally^13,14^ necessitates new and renewed consideration of management effectiveness in supporting reef resilience^21^. To this end, we evaluate the contribution of three management approaches to mitigating, if not preventing, COTS population outbreaks, with a view to minimizing ongoing coral loss. Specifically, we assess the relative efficacy of (1) manual control, (2) marine protected areas (MPAs), and (3) water quality improvement in influencing COTS numbers and coral cover during the current outbreaks in the Cairns Sector of the GBR from 2013 to 2017 (Fig. 1; Supplementary Text S1, S2, S3). While manual control programs have been implemented on the GBR since the 1960s^22^, the program examined here combines single-injection methods^10^ with improvements in how manual control is deployed (Supplementary Text S1)^11,12^. The identification of hypothesized drivers of COTS population outbreaks, namely the loss of natural predators that would limit COTS abundance and the increase in nutrient levels enhancing larval recruitment^1^, has informed management action around these issues prior to and during the current outbreak (Supplementary Text S2, S3)^23,24^. In contrast to manual control, both the use of MPAs to enhance predation and water quality improvement to reduce larval survival represent indirect management interventions, working to protect predator communities or to lower COTS recruitment by reducing phytoplankton biomass. Assessment of the relative effectiveness of these three different approaches to COTS management is now possible given that MPAs (i.e. zoning) have been in place since 1981 and were further expanded in 2004^25^, water quality improvement programs have been implemented since the early 2000s^26^, and strategic manual control has been undertaken since July 2013^11^.

Here we ask whether these three management approaches: (1) reduce COTS densities, (2) are effective in keeping densities low, and critically, (3) improve hard coral cover? Direct comparison of these three approaches is not straightforward, however, given the very different temporal and spatial scales on which they are implemented, measured, and on which they operate on COTS population dynamics. Hence, to assess the relative efficacy of zoning and manual control, we use data on COTS abundance from the current COTS Control Program, and coral cover from the Great Barrier Reef Marine Park Authority’s (GBRMPA) Reef Health Impact Surveys (RHIS), collected at 52 sites at 21 reefs located in three different types of spatial zoning in the Cairns Sector from July 2013 to December 2017 (Fig. 1; see “Methods” for more detail). We compare these results with the achievements in water quality improvement and concurrent changes in coral cover at unculled sites in the Cairns Sector up until December 2017, by using publicly available and refereed scientific reports produced by the Queensland and Australian Government, and the Australian Institute of Marine Science (AIMS). Finally, we consider the implications of our results for how COTS Control Programs are designed at a variety of scales. While our work is focused specifically on the GBR, COTS population outbreaks are commonly experienced elsewhere in the Indo-Pacific and our results will have implications for control efforts across the range of *Acanthaster* spp. and other coral predators, e.g. *Drupella * spp.

## Results and discussion

### Does manual control reduce COTS densities?

We first asked whether manual control could control COTS densities at a site. To do this, we examined COTS control data from 52 sites with permanently marked coral monitoring (RHIS) sampling points, where repeated manual control of COTS took place from July 2013 to December 2017. These 52 sites were located at 21 reefs and were distributed across three different management zones (Fig. 1).

**Figure 2:**
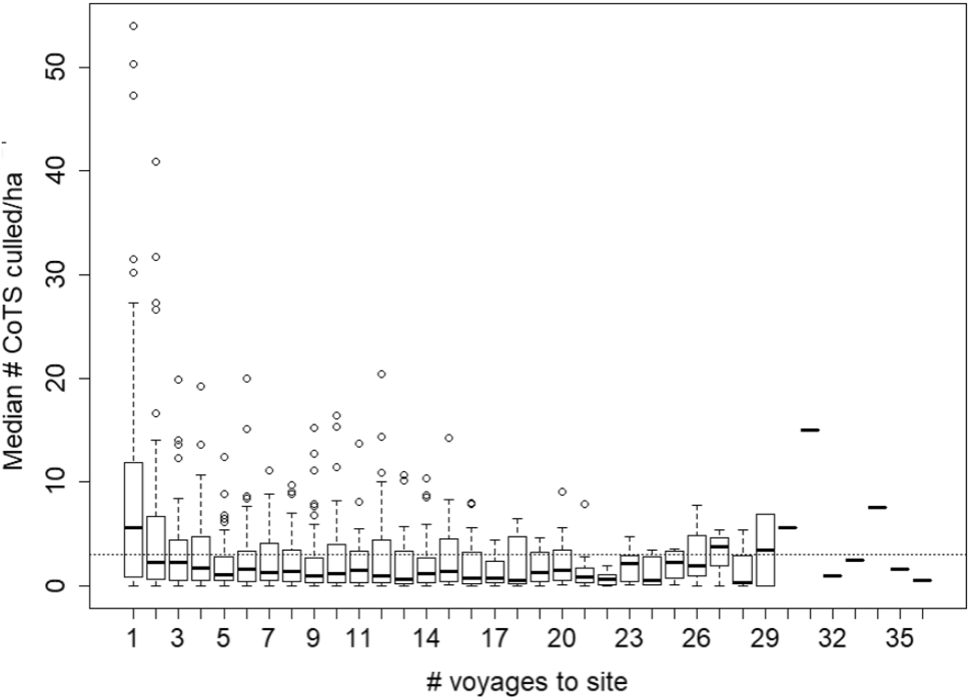
The effect of number of control voyages on the density of Pacific Crown-of-Thorns Starfish (COTS), *Acanthaster* cf*. solaris*. Manual control of COTS took place at a total of 52 sites at 21 reefs in three different types of spatial zoning in the Cairns sector of the Great Barrier Reef from July 2013 to December 2017. The density of COTS encountered during a voyage at a site declined as a function of the number of voyages that had previously visited that site. This decline was initially rapid and after roughly five voyages COTS densities fluctuated while remaining low. Note, beyond 22 visits, sample sizes decline substantially with just six sites visited 23 or more times; variation in COTS densities increases dramatically as a result. Dashed line = the ecologically sustainable threshold for COTS outbreaks^27^, i.e. the density of COTS that can be sustained before coral cover is lost, Solid bar = median, box = quartiles, whiskers = extremes, circles = outliers. Sample sizes given above the x-axis*.*

Over the 4.5 year period, individual sites were visited on average 15 ± 6.2 (s.d.) times (range 5–36), with the number of voyages to a site in a year ranging from 0 to 11 (mean 3.2 ± 2.3 s.d. voyages yr^−1^). At the start of the Control Program, COTS densities at the 52 sites averaged 40 ± 54 s.d. individuals ha^−1^ (range 0–237) and were above an ecologically sustainable density threshold of 3 ha^−1^ at 45 sites. We use this threshold as a benchmark since coral growth is outpaced by predation by an ‘average’ COTS population at sites with low coral cover (estimated as 5 ha^−1^ for just the three largest size categories)^27^, and COTS fertilization (and thus reproductive) success increases substantially at densities of ≥ 3 ha^−1^ due to Allee effects ^28^. Manual control was effective in rapidly reducing COTS densities with the median density of COTS encountered being significantly lower on the second and subsequent voyages to a site than on the first voyage (Fig. 2; Friedman’s chi-squared = 9.31, *df* = 1, *P* = 0.0023; Table S1). This decline was initially rapid with one voyage sufficient to bring the median COTS density to below the ecologically sustainable threshold. The 75th percentile of sites reached this threshold following five culling voyages and fluctuated around the threshold until the number of sites in the analysis dropped to below 5 sites at 23 voyages and two and one site at voyages 27 and 29 respectively. Over the period of the study an average of 126 COTS ha^−1^ (range 5–723) were removed from each site.

The second and subsequent voyages to a site resulted in additional COTS being culled indicating the need for repeated visits. This appears to be largely due to the fact that not all COTS present at a site are visible and available to be culled at any one time^29^ and, to a lesser extent, to immigration into controlled sites from adjacent areas (see below). This result emphasizes the need for repeat voyages to a site in order to achieve sustained, reliable reductions to below the ecological threshold.

**Figure 3:**
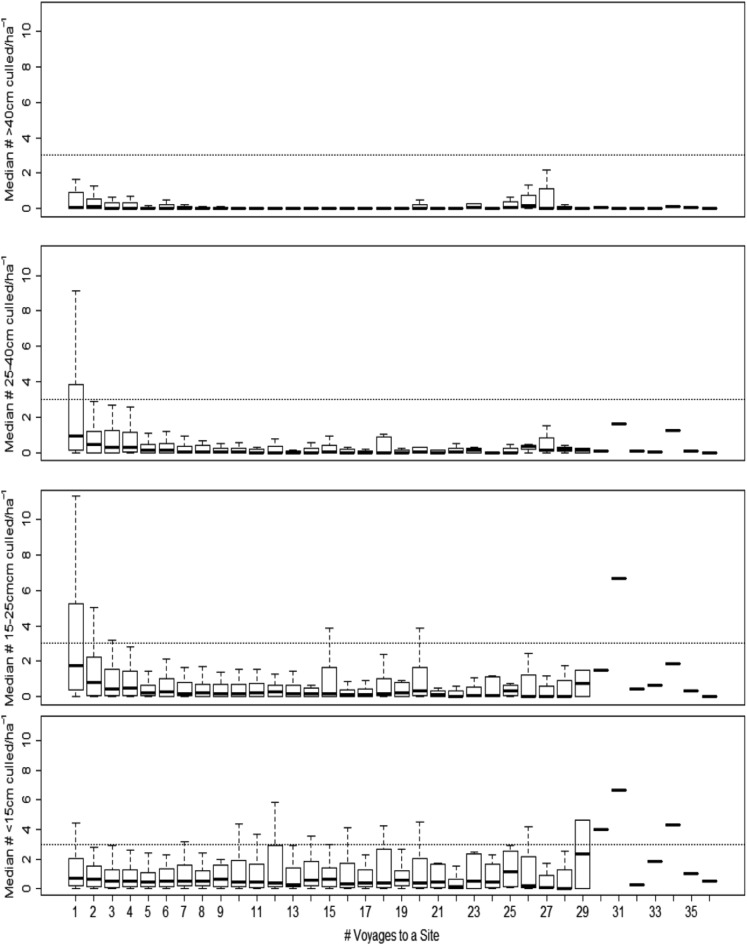
The effect of number of control voyages on the density of Pacific Crown-of-Thorns Starfish (COTS), *Acanthaster* cf*. solaris*, within four size classes. Manual control of COTS took place at a total of 52 sites at 21 reefs in three different types of spatial zoning in the Cairns sector of the Great Barrier Reef from July 2013 to December 2017. The densities of the three largest size classes (> 15–25 cm, > 25–40 cm, and > 40 cm diameter) show a sharp decline during the first four voyages, while densities of the smallest size class (< 15 cm diameter) show a slight and longer-term reduction but remain relatively high. Dashed line = the ecologically sustainable threshold for COTS outbreaks^27^, i.e. the density of COTS that can be sustained before coral cover is lost, Solid bar = median, box = quartiles, whiskers = extremes, circles = outliers. Sample sizes can be found in Fig. 2.

The impact of manual control was not consistent across the four COTS size categories (Fig. 3). The median densities of the largest size classes (> 15–25 cm, > 25–40 cm, and > 40 cm diameter) were significantly lower on the second and subsequent voyages than on the first voyage (Friedman’s chi-squared = 27, *df* = 1, *P* < 0.0000003) and reduced to levels below the ecological threshold (Fig. 3). While the densities of the smallest size class (< 15 cm diameter) also declined after the first voyage, this was to a smaller extent (Fig. 3, lower panel; Friedman’s chi-squared = 9.0, *df* = 1, *P* < 0.0027; Table S1). This might be due to smaller individuals being (1) harder to find, as reported by divers, (2) more likely to emerge in the absence of adults^30^, or (3) more nocturnal and thus less exposed to culling^31^. These results suggest two things. First, manual control effectively targets the most damaging individuals. Because an individual’s coral consumption^32^ and fecundity^33,34^, and therefore its contribution to population dynamics and the potential for irruptions^28^, increases with its size, removing larger individuals from the population is important. The sooner these larger individuals are removed, the greater the reduction in coral loss at the site will be, and, the greater the reduction in the site’s contribution to downstream dynamics and impact will be. Second, the fact that, after just a small number of voyages, larger individuals had been removed from a site and that, thereafter, most individuals culled were from the smaller and harder to find size classes, points to generally low levels of immigration to sites post-control.

Combined, these findings indicate that strategic manual control at specific management locations removed disproportionate numbers of the larger, more fecund, and more damaging COTS, and was effective in keeping COTS densities below the ecologically sustainable level after five or more voyages to a site. This differential pattern of removal of COTS of different sizes suggests that rapid re-visitation in the initial phases of control is key to minimizing damage caused by these larger animals. Over the longer term, less frequent but regular visitation would be required to remove recruiting and immigrant individuals. Realizing these benefits will be most efficiently achieved by balancing re-visitation intervals to the minimum period that optimizes balance between the availability COTS and the economics of re-visitation. The fact that no COTS are available at a site at the end of a voyage but are available on subsequent voyages (an interval of ≥ 7 days) suggests that it is cycles in COTS behaviour, e.g. phases of active foraging and resting^31^, that is influencing their availability at short and long timeframes.

### Manual control improves hard coral cover

The ultimate objective of COTS control is not to kill starfish but rather, to protect live hard coral. Consequently, an important measure of the effectiveness of a control program is the response of hard coral to control efforts. Our results indicate that manual control of COTS was effective in achieving this goal. At the start of the Control Program in July 2013, average hard coral cover at the 52 sites was 26.8% ± 12 s.d. During the 4.5 years period, average coral cover increased by 17.6% ± 85 s.d., with hard coral cover increasing at 25 sites (range: 0.35% to 305%) and decreasing at 27 sites (range: -85% to -3.2%). Specifically, hard coral cover in the last voyage that a site was visited was significantly and positively related to the number of control voyages to have visited that site previously (linear regression; R^2^ = 0.17, F_1, 50_ = 10.08, *P* < 0.003, Table S2). This response in hard coral cover is not explained by sites with higher initial hard coral cover being visited more frequently by the Control Program (*P* = 0.73). In contrast, percent hard coral cover at fixed sites not receiving control (surveyed as part of the AIMS’ Long-Term Monitoring Program) decreased to “historical lows”^35^ (see also Figure a) Benthic cover from fixed survey sites, Hard coral in^35^) in the same period, with these declines being at least in part attributed to the current COTS population outbreaks^36^. Interestingly, in reporting on inshore coral reef surveys, Thompson, et al.^37^ noted that ongoing manual control of COTS at the Frankland Islands between January 2017 and March 2018 had contributed to mitigating their impacts on coral loss.

**Figure 4:**
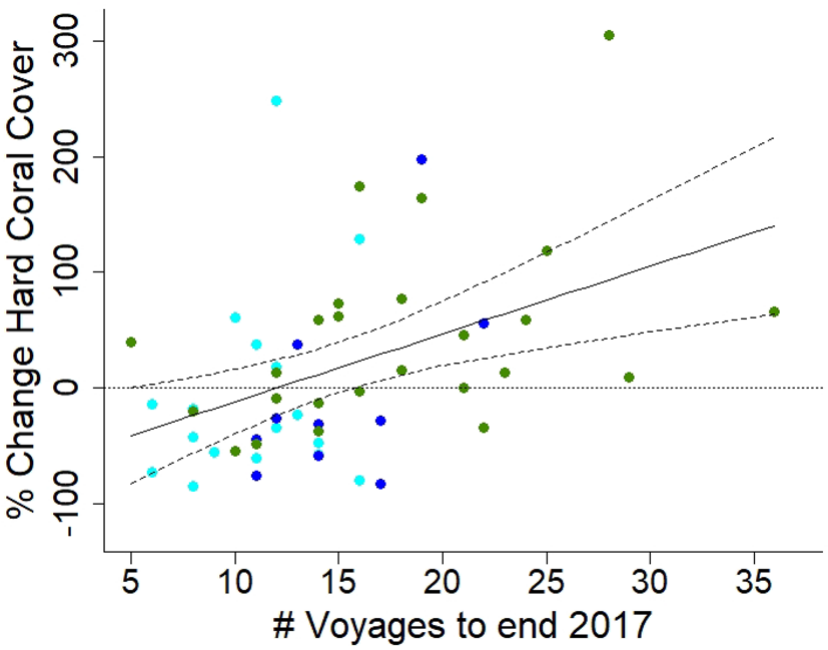
The effect of number of control voyages on the percentage change in hard coral cover to the end of 2017. Manual control of COTS took place at a total of 52 sites at 21 reefs in three different types of spatial zoning in the Cairns sector of the Great Barrier Reef from July 2013 to December 2017. The proportional change in hard coral cover at these 52 sites over the 4.5 year control period was significantly and positively related to the number of voyages that visited these sites. That is, coral cover was not just maintained but actually increased as the number of voyages increased. The horizontal dotted line represents no change in coral cover, the solid line the regression equation (R^2^ = 0.19, F_1, 50_ = 11.99, *P* < 0.0011) and the dashed lines are the 95% confidence limits. Colours indicate zoning: light blue = take zones, dark blue = limited take, green = no take.

Not only was the final absolute hard coral cover related to the effort invested in manual control at a site but the proportional change in hard coral cover at a site relative to its initial cover across the 52 sites over the 4.5 years period was significantly and positively related to the number of voyages that visited these sites (linear regression: R^2^ = 0.19, F_1, 50_ = 11.99, *P* < 0.0011; Fig. 4, Table S2). That is, coral cover was not just maintained but actually improved as the number of control voyages increased. These findings indicate that strategic manual control at specific management locations resulted in an average improvement in hard coral cover, with the proportional change in hard coral cover increasing with the number of control visits to a site.

Though the effect of repeated manual control on live hard coral cover was significant, the amount of variation in the change in hard coral cover relative to initial cover it explained, ~ 20%, could be considered relatively low. That it is of this order of magnitude, however, is not surprising as a multitude of factors, in addition to and independent of COTS predation, influence coral cover dynamics at a site on the GBR^15,38^. De'ath, et al.^15^ estimated that coral predation by COTS was responsible for 42% of the 50% decline in coral cover across the GBR over a 27-year period. This provides us with an initial upper estimate of the magnitude of the effect we might expect from manual control. During this study, two mass bleaching events occurred in 2016 and 2017^16,39^ with severe, though spatially uneven impacts on coral cover in the Cairns Sector of the GBR^18^. It is reasonable to expect that these additional, non-COTS related mortality factors would have had a significant influence on coral cover at the 52 sites during our 4.5 year study and would have limited the amount of variation available to be explained by manual control. Despite the operation of significant non-COTS drivers of hard coral cover during this study, the signal of the effect of manual control of COTS control persisted. This indicates that its effect is strong with manual control significantly improving outcomes for hard coral during a COTS population outbreak and multiple mass bleaching events.

### Zoning influences initial COTS densities

**Figure 5:**
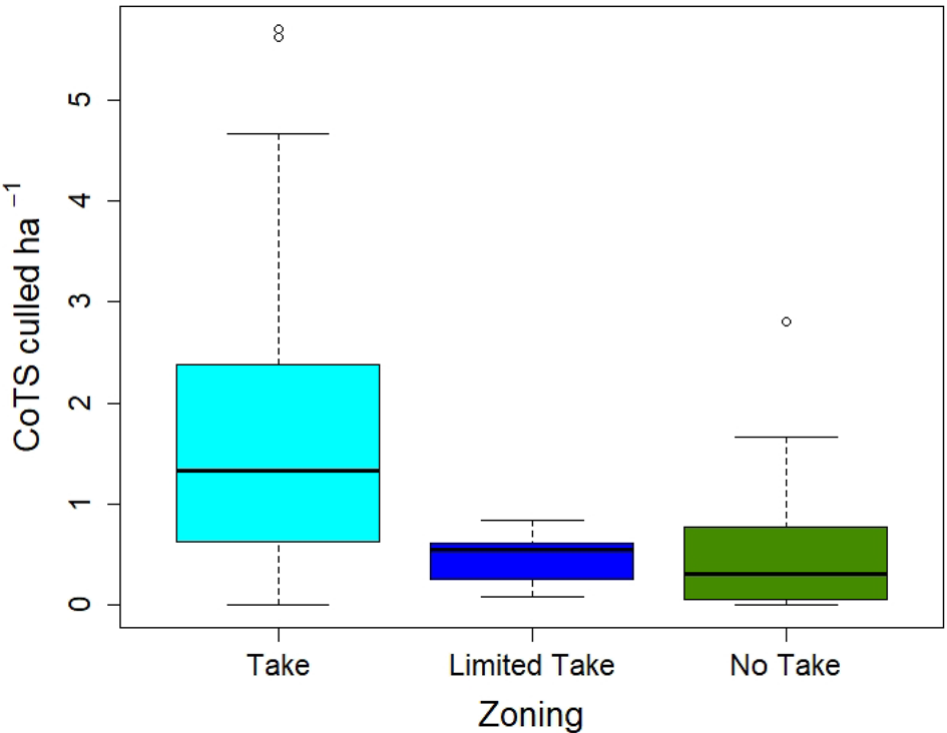
Median densities of COTS culled ha^−1^ when control began at site zoned with different levels of protection. Colours indicate zoning: light blue = take zones, dark blue = limited take, green = no take. Solid bar = median, box = quartiles, whiskers = extremes, circles = outliers.

The 52 sites at which manual control of COTS took place were located in three different management zones, namely in Marine National Park (i.e. ‘no-take’) zones where extractive use is prohibited (n = 26 sites); in Conservation Park (i.e. ‘limited-take’) zones where limited fishing (excluding gill netting and trawling) and collecting are permitted (n = 10); and in Habitat Protection (i.e. ‘take’) zones where fishing and other harvest activities are permitted with the exception of trawling (n = 17) (see “Methods—effect of marine protected areas” for more detail). Our initial analysis showed that at the start of the Control Program in July 2013, hard coral cover did not differ among differently zoned sites (Welch one-way test, F_2,23.78_ = 0.99, *P* = 0.383). In contrast, zoning did influence the density of COTS culled on the first voyage to a site (Welch one-way test, F_2,29.6_ = 5.95, *P* < 0.007, Fig. 5), with a higher COTS density in ‘take’ zones than in ‘limited-take’ or ‘no-take’ zones (Games Howell post-hoc comparisons, *P* = 0.008 and *P* = 0.008, respectively). In addition, the number of control voyages to a site was not independent of zoning (Welch one-way test, F_2,28.92_ = 8.65, *P* = 0.0011), with sites located in ‘take’ zones tending to be visited less frequently than sites located in ‘limited-take’ or ‘no-take’ zones (Games Howell post-hoc comparisons, *P* = 0.012 and *P* = 0.001, respectively). Given this effect of zoning on the initial COTS densities encountered at a site (a dependent variable in our analyses) and on frequency of visitation to a site (an independent variable in our analyses), further analyses examining the combined effects of manual control and of zoning on changes in COTS densities and in hard coral cover was conducted.

### Combined effects of manual control and zoning on hard coral cover

The combined effects of manual control and zoning on hard coral cover were examined for both the final coral cover at a site and for the proportional change in hard coral cover at a site (i.e. final hard coral cover as a proportion of the initial hard coral cover). First, absolute hard coral cover in the last voyage that a site was visited during the 4.5 year period was influenced by initial hard coral cover (β = 0.04, *P* = 0.005) and the number of voyages to visit the site (β = 0.08, *P* = 0.029), while the influence of increased protection through zoning reached only a trend (β = 0.45, *P* = 0.063) (fixed effects model: R^2^ = 0.37, F_3,48_ = 9.455, *P* < 0.0005; Table S3). Second, proportional change in hard coral cover over the 4.5 year period was not influenced by the zoning of a site (β = 0.06, *P* = 0.32), but did increase with the number of voyages to visit the site (β = 0.022, *P* = 0.015) (fixed effects model: R^2^ = 0.21, F_2,50_ = 6.49, *P* = 0.003; Table S3).

Zoning has been linked to a range of ecosystem benefits on the GBR^40–42^, as well as to the potential of a reef to experience a COTS outbreak^43–45^. Our results provide some support for this conclusion: sites zoned with greater protection, i.e. Marine National Parks and Conservation Park zones, had lower COTS densities than sites zoned Habitat Protection at the start of the manual control. Zoning showed a near-significant and positive effect on hard coral cover at the end of the study but was not a significant predictor of the proportional change in hard coral cover. In short, while zoning contributed to the initial conditions at a site, its contribution over the period of the Control Program was small relative to that of manual control. These results, and those of previous studies, suggest that current zoning arrangements act a means of moderating the impact of an active COTS outbreak, and that its role in a COTS Control Program will be as a complementary action used to support manual control or where manual control cannot be employed. The management utility of observed zoning effects on COTS populations is also compromised by the current lack of a mechanistic understanding of how such effects actually occur; whether directly through predation by targeted fish species^46^ or indirectly through cascading trophic or behavioural effects, and whether the main effects are on the pelagic or settled phase or both^34,47^. Determining the role of MPAs, and, in particular, the level of take of various fisheries on the GBR, on COTS densities and population outbreaks is a focus of current work. Finally, it should be remembered that current zoning arrangements were not designed with consideration of any influence of the spatial configuration of MPAs on COTS population outbreaks. Hence, effects of MPAs may well be much larger if designed with COTS outbreaks in mind, e.g. by protecting reefs that are identified as key nodes in COTS outbreak and spread processes^48, 49^.

### Efficacy of water quality improvement on COTS and hard coral cover

Finally, we compare our results against the relative efficacy of water quality improvement in controlling CoTS population outbreaks across the GBR and on reefs in the Cairns Sector up until December 2017. We first examine the scientific evidence for improvements in the quality of water flowing from the catchments adjacent to the GBR as a whole because water quality in the Cairns Sector is influenced by discharge from catchments well outside its boundaries^50^. Progress towards improving GBR water quality, based on scientific monitoring and modelling published in peer-reviewed technical reports^51^, has been reported upon annually since 2011 (Table S4a,b, S5)^52–58^. The most recent GBR Report Card 2019^58^ reports that none of the main agricultural land uses (sugar cane, grazing, horticulture, grain) have achieved their 2018 target to manage 90% of land under best management practice (Table S4a). The reported improvements in agricultural best management practice systems were used to model estimates of the long-term annual river load reductions from 2009 to 2018^58^. These estimates showed that the 2018 water quality targets for each of the key pollutants of concern, including those thought to influence COTS outbreaks (fine sediment, dissolved inorganic nitrogen, particulate nitrogen, particulate phosphorus) have also not been achieved (Table S4b). The marginal reductions in river pollutant loads are reflected in the lack of improvements, and in some cases further decline, in measured water quality along the Wet Tropics coast (adjacent to the Cairns Sector) since 2005 (see Sect. 5.2 and Fig. 5–31, 5,35, and 5–41 in^59^). In particular, trends in chlorophyll-*a* concentrations have been relatively stable and are currently at, or slightly exceeding, the current water quality guideline values^60^. Finally, since 2009 the overall score for water quality on the GBR given in the seven annual report cards (based on eReefs coupled hydrodynamic-biogeochemical model in the most recent report cards; see Supplementary Material Text S3), has fluctuated between ‘poor’ and ‘moderate’ (Table S6)^52–58^. Combined, these measured and modelled results point to slow, if any, progress having been made in achieving GBR water quality improvement, including in the Cairns Sector^26,61^.

The lack of meaningful improvement, and in some cases further decline in measured GBR water quality since the implementation of various Reef Plans starting in 2003^58,59^, mean that the efficacy of water quality improvements in reducing COTS population outbreaks and their impacts on hard coral cover are likely to be negligible. This is borne out for the Cairns Sector and the GBR generally by the fact that the current COTS population outbreak began in 2010^1^, well after implementation of water quality improvement programs commenced in 2003, and has since moved from further north, through the Cairns (this study) and adjacent Sectors, southward along the GBR^36,37^. Not only did water quality improvement not prevent a COTS outbreak but hard coral cover in the study region showed no improvement after its implementation. The AIMS Long Term Monitoring Program reports that the trend in hard coral cover in the Cairns Sector at sites not receiving COTS control was a decline to “historical lows” (Figure a) Benthic cover from fixed survey sites, Hard coral ^35^) in the period of this study. Moreover, predation by COTS had contributed to reductions in coral cover on some inshore reefs in the Wet Tropics region from 2012 to 2017^37^.

These findings indicate there is little reason to expect that water quality improvement efforts have acted to suppress COTS population dynamics, or that any such influence would be detected at this point in time. The reductions in river pollutant loads (Table S4b) are sufficiently marginal to suggest that recent efforts to reduce land-based pollution (Table S4a) are unlikely to protect GBR ecosystems from declining water quality^26,62^, including lowering phytoplankton biomass and associated recruitment of COTS larvae^1^. This becomes particularly evident when compared with the magnitude of change in land use and management required to obtain substantial reductions in river pollutant loads to coastal receiving waters from international examples where measurable improvements in coastal water quality have been achieved^63^. Hence, while water quality improvement may ultimately prove efficacious in influencing COTS population dynamics and outbreaks, it cannot yet be solely relied upon for COTS control in the GBR. Finally, further field observations linking larval abundance using eDNA^64^ and environmental factors, combined with laboratory experiments on the effects of different nutrient and feeding regimes on juvenile COTS condition^65^, are needed to elucidate the role of catchment-derived nutrients in driving COTS population outbreaks.

## Conclusion

In the face of increasing and elevated COTS densities on reefs on the GBR, and the Indo-Pacific more generally, the effectiveness of different management approaches in mitigating COTS population outbreaks needs to be assessed, particularly in the context of enhancing reef resilience given the sustained and ongoing degradation of coral reefs globally^13,14^. Our comparison of the three management approaches to COTS control used on the GBR to date suggests the following three conclusions. First, there is little evidence that the water quality improvement efforts have resulted in detectable changes in water quality on the GBR to date. While sustained action to improve water quality will likely have important and far reaching benefits for the condition and resilience of the GBR, the failure of efforts to date in achieving measurable improvements means that water quality interventions cannot currently be solely relied upon for reducing COTS densities or population outbreaks. Second, the initial conditions reported from a site were influenced by the extent to which it was protected from fishing; sites afforded the greatest protection (i.e. zoned Marine National Park and Conservation Park) had fewer COTS at the outset than did less protected sites (i.e. those zoned Habitat Protection). The influence of zoning on COTS densities, however, was not significant at the end of the Control Program. Third, not only was strategic and repeated manual control effective in reducing overall COTS densities and in skewing the population size structure towards smaller, less damaging individuals, but in doing so it allowed recovery of hard coral cover, thereby directly achieving the ultimate goal of COTS control.

Given the outcomes for hard coral cover reported here, the Control Program must be viewed as an important tool in the context of managing the resilience of the GBR in the face of global environmental change, and, will likely remain so for some time. As such, it stands alongside and supports other local management measures considered essential to reduce hard coral loss and conserve coral reef functionality in the hope that global warming can eventually be contained. For example, recent modelling suggests that without COTS control management interventions such as coral restoration using transplanted and engineered corals are unlikely to be successful^66^. Until the effects of global environmental change can be stemmed and reversed, the GBR is likely to continue to experience frequent bleaching, such as the episodes in 2016 and 2017^14^ and predation of corals by COTS under such conditions is certain to further reduce the system’s overall resilience. Hence, manual control currently represents the only demonstrably effective means of addressing the threat posed by COTS and will continue to be a key tool in supporting reef resilience into the future.

## Methods

### Study area

The GBR extends over 2,000 km along the northeast coast of Australia, covering an area of ~ 348,000 km^2^ including ~ 20,000 km^2^ of coral reefs^67^. This study focuses on reefs in the Cairns Sector and in particular on 52 sites at 21 reefs where both strategic manual control and Reef Health Impact Surveys (RHIS) were conducted from July 2013 to December 2017 (Fig. 1). These sites and reefs were considered to be either economically or ecologically important (see “Effect of manual control” below) and, were subjected to three different levels of protection (see “Effect of marine protected areas” below). The catchments discharging into and influencing water quality in the Cairns Sector, including the Barron, Mulgrave-Russell, Johnstone, Tully, Herbert and Burdekin rivers, have been the focus of long-term and extensive water quality improvement efforts^68^ (see “Effect of water quality improvement”).

### Effect of manual control

Data on COTS densities at the 52 control sites came from the COTS Control Program. Funded by the Australian Government through the GBRMPA (Supplementary Text S1), the program’s on water operations were conducted by the Association of Marine Park Tourism Operators (AMPTO) using dedicated control vessels crewed by specially trained and experienced COTS control divers. Sites selected for manual control were either sites that were economically important for tourism, or located on reefs that oceanographic modelling suggested were highly connected to other reefs and, therefore, potentially significant in the pattern of coral and COTS larval spread^48^. During the period covered by this study, sites and the pattern of visitation were chosen by operators and the GBRMPA based on factors such as operational considerations, time since last visit, and reports of COTS. Decisions were made annually with some modification during the year. The sites included in this study were a sub-set of the total number of sites and included all sites which had a permanent survey marker. This enabled accurate relocation of RHIS survey sites for repeated measures of coral cover throughout the study period.

COTS control vessels undertook 10-day control voyages, during which selected reefs and specific sites on those reefs, were dived. Each of the 52 sites included in this analysis were GPS mapped polygons with an average size of 14 ha (± 13.6 s.d.). Each site was thoroughly searched on SCUBA and COTS were culled until no more were available. When densities were high this required multiple dives, by multiple divers, during a single voyage. Divers kept a tally of the number of COTS killed during each dive and the total number of COTS removed from each site during a voyage was calculated as the sum of these tallies across dives and divers at a site.

In our analysis, we use the number of voyages to visit a site as our metric for control effort. We do this because, during a voyage, dives continued at a site until COTS were no longer available to cull. Thus, the voyage represents a standardized unit of management outcome at a site.

We used a COTS density of 3 ha^−1^ as our success threshold as this value was conservative estimate of a density above which hard coral growth is estimated to be outpaced by predation (which based on the three largest size classes is 5 COTS ha^−1^ at 20% coral cover)^27^ and the density at which fertilization, and therefore reproductive, success is maximized (3 COTS ha^−1^)^28^. Sites where manual control reduced and maintained COTS densities to below this threshold were sites where control was considered successful.

### Reef health impact surveys

RHIS surveys are the standardized survey protocol used by the GBRMPA for monitoring coral condition on the GBR^67,69^. RHIS surveys plots are circular with a radius of 5 m. Within these plots observers estimate a range of coral health indicators including hard coral cover and the presence and extent of a range of impacts, including COTS numbers. The method has been shown to be robust and an effective means of assessing coral health^69^.

In this study data on coral cover at the 52 control sites came from RHIS surveys conducted by trained and experienced observers who were either employees of the COTS Control Program or of the GBRMPA’s Joint Field Management Program. At each site, three RHIS survey locations were distributed at roughly equal distances across the site resulting in a total survey area per cull site of 235m^2^. Each RHIS point was permanently marked with a steel picket to ensure the same area was surveyed in each survey. Percentage hard coral cover was visually estimated and the percentage hard coral cover for each site was calculated as the mean percent hard coral cover of the three RHIS plots at the site.

### Effect of marine protected areas

The zoning, or degree of protection, for each of the sites (see “Effect of manual control” below) was determined by reference to the GBRMPA’s zoning maps (Supplementary Text S2)^70^. Twenty-six of the 52 sites were classified as Marine National Park or ‘no-take’ zones where extractive use is prohibited, ten were classified as Conservation Park where limited fishing (excluding gill netting and trawling) and collecting are permitted, and the remaining 17 were zoned as Habitat Protection where fishing and other harvest activities are permitted with the exception of trawling^25^. For the purposes of this study we refer to these three zoning categories as ‘no-take’, ‘limited-take’ and ‘take’ zones, respectively. To examine the effect of zoning type on COTS numbers and coral cover at the 52 sites in the Cairns Sector, we included zoning category as a co-variable in our analyses (see “Data analyses” below).

### Data analyses

Our data analyses focused on determining whether the strategic manual control program could have influenced COTS densities and coral cover at individual sites, alone and in combination with zoning. We used ANOVA and linear mixed models to assess the influence on COTS densities of both zoning and manual control. Mean hard coral cover at a site and change in hard coral cover between the first and last year a site was visited expressed as a percentage of the initial hard coral cover, were the dependent variables. A site’s zoning and the total number of voyages received were included as independent variables.

Analyses were conducted using the R statistical software^71^. Assumptions for each analysis were tested as appropriate. Normal Q-Q plots were used to assess normality and the dependent variable was square-root transformed when necessary to meet this assumption. Visual inspection of the distribution of residuals relative to fitted values and their leverage was used to assess assumptions of skewness, kurtosis, homoscedasticity and the performance of the link function. These assessments were then confirmed using the *gvlma* package^72^ in R. In the linear mixed models, potential spatial dependence between sites at the same reef was accounted for by including the variable ‘reef’ as a random effect with the other dependent variables included as fixed effects. The *lme4* package^73^ was used for linear mixed models, and the *lmerTest* package^74^ was used to test the significance of random and fixed effects. The random effect ‘reef’ was not significant in any of the models and fixed effect models were ultimately used.

### Effect of water quality improvement

To examine the effectiveness of management actions around water quality improvement (Supplementary Text S3) to reduce COTS recruitment and population outbreaks, we have relied on the annual report cards published by the Queensland and Australian governments since 2011^52–58^, and the associated publicly-available scientific and technical publications on which they are based, e.g.,^37,59^. These annual report cards measure progress towards the goals and associated water quality targets and land management and catchment targets set for 2018 and 2020, as outlined in the two Reef Plans that have been implemented to improve GBR water quality since 2009 (Table S4a,b, S5)^26^. To examine potential associated changes in coastal water quality, in COTS densities and population outbreaks on coastal reefs, and in coral cover on coastal reefs in the Cairns Sector, we have relied on reports from the Marine Monitoring Program Inshore Water Quality Monitoring^59^ and Inshore Coral Reef Monitoring^37^.

## Supplementary information

Supplementary information.

## Data Availability

All data needed to evaluate the conclusions in the paper are present in the paper and/or the Supplementary Information. Additional data related to this paper may be requested from the corresponding author.
