# Supplementary material for: Relative efficacy of three approaches to mitigate Crown-of-Thorns Starfish outbreaks on Australia’s Great Barrier Reef
Source: Sci Rep. 2020 Jul 28;10:12594. doi: 10.1038/s41598-020-69466-1 (PMC7387460; doi:10.1038/s41598-020-69466-1)
Supplement: Supplementary file 1 — Supplementary information. [file 41598_2020_69466_MOESM1_ESM.docx]

Supplementary Material

Relative efficacy of three approaches to mitigate Crown-of-Thorns Starfish outbreaks on Australia’s Great Barrier Reef

David A. Westcott^1*^, Cameron S. Fletcher^1^, Frederieke J. Kroon^2^, Russ Babcock^3^, Eva Plagányi^3^, Morgan S. Pratchett^4^, Mary Bonin^5^.

^1^ CSIRO, Land and Water, Atherton, Queensland, 4883, Australia.

^2^ Australian Institute of Marine Sciences, Townsville, Queensland, 4810, Australia.

^3^ CSIRO, Oceans and Atmosphere, PO Box 2538, Brisbane, Queensland, 4072, Australia.

^4^ ARC Centre of Excellence for Coral Reef Studies, James Cook University, Townsville, Queensland, 4810, Australia.

^5^ Great Barrier Reef Marine Park Authority, Townsville, Queensland, 4883, Australia.

* David A. Westcott Corresponding author

**Email:**  david.westcott@csiro.au

Text S1. Manual Control

While water quality improvement measures and zoning act to moderate hypothesized drivers of COTS outbreaks, manual control acts to reduce COTS populations directly. Following the 1960s outbreak, the Australian Academy of Sciences committee responsible for reviewing management responses to the 1960s outbreak to recommend that future controls be conducted only around high-value tourism sites as broad-scale approaches to manual control were not available^1^. In the subsequent two outbreaks on the GBR (beginning 1979 and 1993 respectively) the focus of manual control programs remained at high-value tourism sites. Nevertheless, the prospect of effective manual control capable of reducing COTS densities and improving hard coral cover was not forgotten. In their reviews of manual control programs, Zann and Weaver ^2^ and Birkeland and Lucas ^3^ suggested that the success of manual control programs was inversely related to the scale of the outbreak with which they were concerned. The probability of success further increased with greater warning of an outbreak; when COTS were tightly aggregated and accessible; when COTS populations were small; when lethal injections were used; and when resources were adequate and allowed for repeated control efforts^2,4^. Programs failed when COTS were superabundant, when there was unfettered migration into the control area, and when they were difficult to detect^5^. The overall impression that programs were not successful led to the assumption that manual control methods could not be scaled up to provide effective control at large spatial scales^6^.

This situation changed with two new developments during the current COTS population outbreak on the GBR. The first development was the discovery that a single small volume injection of oxbile was an effective means of killing COTS *in situ*^7^. This method revolutionized manual COTS control by enormously reducing the effort required to kill an individual and bringing the probability of a successful kill to close to 100%. The second development was the move to a strategic manual control program ^8^. While this new approach still incorporated the management of key sites by tourism operators, it added a dedicated control program vessel to supplement those efforts and included additional sites considered to be of high ecological value. This resulted in the employment of a standardized approach and improved data collection across the program to enable a scientific assessment of the program’s effectiveness. The net result has been a manual control program that has focused on key tourism and selected ecologically important sites since July 2013 and throughout the current outbreak period. This program has also acted as the foundation for the development of a more structured Integrated Pest Management approach to COTS control, that offers the potential for achieving meaningful outcomes at the scale of the GBR^8^. To support the implementation of the current manual Control Program, the Australian Government have invested approximately AUS$14M over the period 2012 to 2017, with an additional AUS$57.8M of Federal funding announced for COTS control in 2018^9^.

Text S2. Marine Protected Areas

The loss of predators, i.e. the ‘*predator removal*’ hypothesis, has long been posited as a potential contributor to COTS population outbreaks^10^. Specifically, the severity of COTS outbreaks may be mediated by the combined consumption of COTS at one or more life history stages by a range of predators^10,11^. Predation may impact recruitment rates of COTS larvae into benthic habitats^12^, and may change growth, reproduction or mortality of COTS post-settlement^10,13-15^. A recent review identified 80 species of coral reef organisms, including fishes and invertebrates that predate on different life history stages of COTS^11^. However, only a few field observations exist of consumption of early life history stages and of healthy adults^11,16,17^, at least partially due to the difficulties of observing predation on COTS gametes, larvae, and settled juveniles^18-20^. Despite the paucity of field observations of predation, the ‘*predator removal*’ hypothesis has received support from modelling at least in relation to certain phases of the COTS outbreak cycle^21-23^.

One management approach for conserving marine ecosystems, including predatory species, is through the use of marine protected areas (MPAs)^24^. Marine protected areas have been a key component of the management of the GBR since the introduction of the first zoning plan for the Marine Park in 1981^25^. This plan was revised in 2004, with the no-take zones increasing from 4.5% to 33% of the Marine Park, and the representation of habitat types in the no-take zones improved. The measured benefits of this approach on the GBR range from the increases in stocks of key fisheries species^26^, including potential predators of COTS, through to enhancing ecosystem resilience^25,27-29^. Recent work suggests that fewer COTS are observed in no-take zones^30,31^ and that the impacts of COTS outbreaks were reduced in no-take zones^28^.

Text S3. Water Quality Improvement

One of the foremost hypotheses proposed to account for COTS population outbreaks, the ‘*terrestrial runoff*’ hypothesis, states that high nutrient availability increases phytoplankton biomass, which in turn enhances COTS larval growth and survival leading to mass recruitment events and outbreaks^32-35^. On the GBR, the four recorded primary COTS outbreaks originating in the Lizard Island - Cairns region since the 1960s were preceded by major flooding events in the Burdekin and Wet Tropics rivers^36-38^. Terrestrial sediment and nutrient loads discharged from these rivers into the GBR lagoon have increased significantly since European settlement in the 1850s^39,40^. Recent catchment modelling estimates that river loads of biologically available nutrients, such as dissolved inorganic nitrogen (DIN), have increased 1.2 to 6.0 times relative to their pre-colonization levels^39,40^. Despite ongoing scientific debate about the validity of the ‘*terrestrial runoff*’ hypothesis^41,42^, the hypothesized link between elevated terrestrial runoff and COTS outbreaks has become a central argument for policy and investment to improve GBR water quality^43-46^.

To protect the GBR from diffuse-source pollution from agricultural land uses^47^, the Australian and Queensland governments first released the Reef Water Quality Protection Plan (hereafter ‘Reef Plan’) in 2003 ^48^. While improving the quality of water flowing through the GBR catchment and into the GBR has a range of high value benefits in terrestrial, aquatic and marine realms^45,49,50^, here we focus specifically on anticipated impacts on COTS population dynamics. The 2003 Reef Plan was revised and updated several times including new goals. The 2003 Plan’s goal of halting and reversing the decline in water quality entering the reef within 10 years, was reaffirmed in 2009 with additional goal ensuring water from adjacent catchemtns had no detrimental effect on the GBR. and associated water quality targets and land management and catchment targets for 2018 and 2020 (Tables S4, S5)^49^. To support the implementation of these various Reef Plans, the Australian and Queensland governments have invested approximately AUD$850M over the period 2009 to 2018^50,51^, with an additional AUD$200M of Federal funding announced in 2018^9^.

Progress towards improving GBR water quality against the goals and targets outlined in the Reef Plans 2009 and 2013 has been reported upon annually since 2011^52-58^. The scorings presented in these annual reports are based on scientific monitoring and modelling published in peer-reviewed technical reports from universities, research organisations and Government agencies (see, for example, https://www.reefplan.qld.gov.au/tracking-progress/reef-report-card/methods-to-create-report-card). Monitoring of marine water quality and hard coral cover has been conducted since 2005^59,60^. The findings of these peer-reviewed technical reports are used to derive scorings for progress against goals and targets in the annual report cards, including for marine water quality and coral cover. In addition, the most recent report cards (2017, 2018) also use marine modelling (namely eReefs coupled hydrodynamic - biogeochemical model) to derive scores for marine water quality.

Table S1

| **Cohort** | **Friedman’s Chi-squared** | **Df** | **P value** |
| --- | --- | --- | --- |
| Total COTS ha^-1^ | 9.3077 | 1 | 0.0023 |
| COTS>40cm ha^-1^ | 10.39 | 1 | 0.000942 |
| COTS 25-40 cm ha^-1^ | 25.13 | 1 | 0.00000054 |
| COTS 15-25cm ha^-1^ | 31.39 | 1 | 0.000000022 |
| COTS <15 ha^-1^ | 9 | 1 | 0.0027 |

Table S2

Linear model results for the relationship between the number of voyages to visit a site and i) the final hard coral cover at a site, ii) the change in hard coral cover over the study period, measured as change in hard coral cover expressed as a percentage of initial coral cover.

|  | **Final % hard coral cover** | | | **% Change in hard coral cover** | | |
| --- | --- | --- | --- | --- | --- | --- |
| *Predictors* | *Estimates* | *CI* | *p* | *Estimates* | *CI* | *p* |
| (Intercept) | 3.52 | 2.46 – 4.58 | **<0.001** | 0.62 | 0.38 – 0.87 | **<0.001** |
| # voyages | 0.10 | 0.04 – 0.17 | **0.003** | 0.03 | 0.01 – 0.04 | **0.001** |
| Observations | 52 | | | 52 | | |
| R^2^ / R^2^ adjusted | 0.168 / 0.151 | | | 0.193 / 0.177 | | |

Table S3

|  | **Final % Hard Coral Cover** | | | **% Change in Hard Coral Cover** | | |
| --- | --- | --- | --- | --- | --- | --- |
| *Predictors* | *Estimates* | *CI* | *p* | *Estimates* | *CI* | *p* |
| (Intercept) | 1.33 | -0.15 – 2.82 | 0.078 | 0.50 | 0.14 – 0.85 | **0.007** |
| # voyages | 0.08 | 0.01 – 0.14 | **0.029** | 0.02 | 0.00 – 0.04 | **0.015** |
| Initial coral cover | 0.04 | 0.01 – 0.07 | **0.005** |  |  |  |
| prot | 0.45 | -0.03 – 0.92 | 0.063 | 0.06 | -0.06 – 0.18 | 0.323 |
| Observations | 52 | | | 52 | | |
| R^2^ / R^2^ adjusted | 0.371 / 0.332 | | | 0.210 / 0.177 | | |

Table S4: Progress against (a) Land and Catchment Management Targets, and (b) Water Quality Targets, for Reef Plans 2009 and 2013*

(a)(b)

* Report Cards for 2009 to 2016 based on 2009 baseline; Report Card 2017/2018 based on 2016 benchmark

Table S5 Progress against Marine Water Quality Targets for Reef Plans 2009 and 2013*

* Report Cards for 2009 to 2016 based on 2009 baseline; Report Card 2017/2018 based on 2016 benchmark

References for Supplementary Material

1 Walsh, R. *et al.* Report of the Committee on the problem of the crown-of-thorns starfish (Acanthaster planci L.). 45 (Commonwealth Government Printing Office, Canberra, Australia, 1971).

2 Zann, L. & Weaver, K. in *Proceedings of the 6th International Coral Reef Symposium* Vol. Vol. 2: Contributed Papers (eds J.H. Choat *et al.*) (International Coral Reef Society, 1988).

3 Birkeland, C. & Lucas, J. S. *Acanthaster planci: major management problem of coral reefs*. (CRC press, 1990).

4 Fisk, D. A. & Power, M. C. *Development of cost-effective control strategies for crown-of-thorns starfish*. (CRC Reef Research Centre, 1999).

5 Gladstone, W. in *The Possible cause and consequences of outbreaks of the crown-ofthorns starfish.* (eds Udo Engelhardt & Brian Lassig) 147 - 156 (Great Barrier Reef Marine Park Authority, Proceedings of a workshop held in Townsville, Queensland, Australia, 1993).

6 Kenchington, R. & Kelleher, G. Crown-of-thorns starfish management conundrums. *Coral Reefs* **11**, 53-56, doi:10.1007/bf00357422 (1992).

7 Rivera-Posada1, J., Caballes, C. F. & Pratchett, M. S. Size-related variation in arm damage frequency in the crown-of-thorns sea star, Acanthaster planci. *JCLM*, doi:10.12980/jclm.2.2014j52 (2014).

8 Westcott, D. A., Fletcher, C. S., Babcock, R. & Plaganyi-Lloyd, E. A Strategy to Link Research and Management of Crown-of-Thorns Starfish on the Great Barrier Reef: An Integrated Pest Management Approach. Report to the National Environmental Science Programme. . 77 (Cairns, 2016).

9 Great Barrier Foundation. Investment Strategy Executive Summary. 4 (Brisbane, Queensland, 2019).

10 Endean, R. Report on investigations made into aspects of the current Acanthaster planci (crown of thorns) infestations of certain reefs of the Great Barrier Reef. (Queensland Department of Primary Industries (Fisheries Branch), Brisbane, Australia, 1969).

11 Cowan, Z.-L., Pratchett, M., Messmer, V. & Ling, S. Known Predators of Crown-of-Thorns Starfish (Acanthaster spp.) and Their Role in Mitigating, If Not Preventing, Population Outbreaks. *Diversity* **9**, 7 (2017).

12 Cowan, Z.-L., Dworjanyn, S. A., Caballes, C. F. & Pratchett, M. Benthic Predators Influence Microhabitat Preferences and Settlement Success of Crown-of-Thorns Starfish (Acanthaster cf. solaris). *Diversity* **8**, 27 (2016).

13 Antonelli, P. L. & Kazarinoff, N. D. Starfish predation of a growing coral reef community. *Journal of Theoretical Biology* **107**, 667-684, doi:http://dx.doi.org/10.1016/S0022-5193(84)80138-1 (1984).

14 Dana, T. F., Newman, W. A. & Fager, E. W. Acanthaster Aggregations: Interpreted as Primarily Responses to Natural Phenomena ﻿. *Pacific Science* **26**, 355 - 372 (1972).

15 Ormond, R. *et al.* in *Acanthaster and the Coral Reef: A Theoretical Perspective* Vol. 88 *Lecture Notes in Biomathematics* (ed Roger Bradbury) Ch. 12, 189-207 (Springer Berlin Heidelberg, 1990).

16 Messmer, V., Pratchett, M. & Chong-Seng, K. Variation in Incidence and Severity of Injuries among Crown-of-Thorns Starfish (Acanthaster cf. solaris) on Australia’s Great Barrier Reef. *Diversity* **9**, 12 (2017).

17 Keesing, J. K., Halford, A. R. & Hall, K. C. Mortality rates of small juvenile crown-of-thorns starfish Acanthaster planci on the Great Barrier Reef: implications for population size and larval settlement thresholds for outbreaks. *Marine Ecology Progress Series* **597**, 179-190, doi:10.3354/meps12606 (2018).

18 Lucas, J. S. Growth, maturation and effects of diet in *Acanthasterplanci* (L.) (Asteroidea) and hybrids reared in the laboratory. *Journal of Experimental Marine Biology and Ecology* **79**, 129-147, doi:10.1016/0022-0981(84)90214-4 (1984).

19 Yokochi, H. & Ogura, M. Spawning period and discovery of juvenile Acanthaster planci (L.) (Echinodermata: Asteroidea) at northwestern Iriomote-jima, Ryukyu Islands. *Bulletin of Marine Science* **41**, 611–616 (1987).

20 Zann, L., Brodie, J., Berryman, C. & Naqasima, M. Recruitment, ecology, growth and behavior of juvenile Acanthaster planci (L.) (Echinodermata: Asteroidea). *Bulletin of Marine Science* **41**, 561–575 (1987).

21 McCallum, H. I. Predator regulation of Acanthaster planci. *Journal of Theoretical Biology* **127**, 207-220, doi:http://dx.doi.org/10.1016/S0022-5193(87)80131-5 (1987).

22 McCallum, H. I. in *Acanthaster and the Coral Reef: A Theoretical Perspective* Vol. 88 *Lecture Notes in Biomathematics* (ed Roger Bradbury) Ch. 13, 208-219 (Springer Berlin Heidelberg, 1990).

23 Morello, E. B. *et al.* Model to manage and reduce crown-of-thorns starfish outbreaks. *Marine Ecology Progress Series* **512**, 167-183, doi:10.3354/meps10858 (2014).

24 Lester, S. E. *et al.* Biological effects within no-take marine reserves: a global synthesis. *Marine Ecology Progress Series* **384**, 33-46 (2009).

25 McCook, L. J. *et al.* Adaptive management of the Great Barrier Reef: A globally significant demonstration of the benefits of networks of marine reserves. *Proceedings of the National Academy of Sciences* **107**, 18278-18285, doi:10.1073/pnas.0909335107 (2010).

26 Emslie, Michael J. *et al.* Expectations and Outcomes of Reserve Network Performance following Re-zoning of the Great Barrier Reef Marine Park. *Current Biology* **25**, 983-992, doi:https://doi.org/10.1016/j.cub.2015.01.073 (2015).

27 Yates, P. M., Tobin, A. J., Heupel, M. R. & Simpfendorfer, C. A. Benefits of marine protected areas for tropical coastal sharks. *Aquat. Conserv.-Mar. Freshw. Ecosyst.* **26**, 1063-1080, doi:10.1002/aqc.2616 (2016).

28 Mellin, C., MacNeil, M. A., Cheal, A. J., Emslie, M. J. & Caley, M. J. Marine protected areas increase resilience among coral reef communities. *Ecology Letters* **19**, 629-637, doi:10.1111/ele.12598 (2016).

29 Castro-Sanguino, C. *et al.* Detecting conservation benefits of marine reserves on remote reefs of the northern GBR. *Plos One* **12**, 24, doi:10.1371/journal.pone.0186146 (2017).

30 Sweatman, H. No-take reserves protect coral reefs from predatory starfish. *Current Biology* **18**, R598-R599, doi:http://dx.doi.org/10.1016/j.cub.2008.05.033 (2008).

31 Vanhatalo, J., Hosack, G. R. & Sweatman, H. Spatiotemporal modelling of crown-of-thorns starfish outbreaks on the Great Barrier Reef to inform control strategies. *Journal of Applied Ecology*, n/a-n/a, doi:10.1111/1365-2664.12710 (2016).

32 Birkeland, C. Terrestrial runoff as a cause of outbreaks of Acanthaster planci (Echinodermata: Asteroidea). *Marine Biology* **69**, 175-185, doi:10.1007/BF00396897 (1982).

33 Fabricius, K. E., Okaji, K. & De’ath, G. Three lines of evidence to link outbreaks of the crown-of-thorns seastar Acanthaster planci to the release of larval food limitation. *Coral Reefs* **29**, 593-605, doi:10.1007/s00338-010-0628-z (2010).

34 Wolfe, K., Graba-Landry, A., Dworjanyn, S. A. & Byrne, M. Larval Starvation to Satiation: Influence of Nutrient Regime on the Success of Acanthaster planci. *Plos One* **10**, 17, doi:10.1371/journal.pone.0122010 (2015).

35 Brodie, J., Devlin, M. & Lewis, S. Potential Enhanced Survivorship of Crown of Thorns Starfish Larvae due to Near-Annual Nutrient Enrichment during Secondary Outbreaks on the Central Mid-Shelf of the Great Barrier Reef, Australia. *Diversity* **9**, 17 (2017).

36 Wooldridge, S. A. & Brodie, J. E. Environmental triggers for primary outbreaks of crown-of-thorns starfish on the Great Barrier Reef, Australia. *Marine Pollution Bulletin* **101**, 805-815, doi:https://doi.org/10.1016/j.marpolbul.2015.08.049 (2015).

37 Brinkman, R., Tonin, H., Furnas, M., Schaffelke, B. & Fabricius, K. Targeted analysis of the linkages between river runoff and risks for crown-of-thorns starfish outbreaks in the Northern GBR. 47 (Australian Institute of Marine Science, Townsville, Australia, 2014).

38 Furnas, M., Brinkman, R., Fabricius, K., Tonin, H. & Schaffelke, B. in *Assessment of the relative risk of water quality to ecosystems of the Great Barrier Reef: Supporting Studies. A report to the Department of the Environment and Heritage Protection, Queensland Government, Brisbane.* 229 (Tropwater, 2013).

39 Bartley, R. *et al.* in *Scientific Consensus Statement 2017: A synthesis of the science of land-based water quality impacts on the Great Barrier Reef* (State of Queensland, 2017).

40 Kroon, F. J. *et al.* River loads of suspended solids, nitrogen, phosphorus and herbicides delivered to the Great Barrier Reef lagoon. *Marine Pollution Bulletin* **65**, 167-181, doi:https://doi.org/10.1016/j.marpolbul.2011.10.018 (2012).

41 Pratchett, M. S., Caballes, C. F., Rivera-Posada, J. A. & Sweatman, H. P. A. Limits to Understanding and Managing Outbreaks of Crown-of-Thorns Starfish (*Acanthaster* spp.). *Oceanography and Marine Biology: An Annual Review* **52**, 133-200, doi:doi:10.1201/b17143-410.1201/b17143-4 (2014).

42 Pratchett, M. *et al.* Thirty Years of Research on Crown-of-Thorns Starfish (1986–2016): Scientific Advances and Emerging Opportunities. *Diversity* **9**, 41 (2017).

43 Roth, C. *et al.* Reef 2050 Plan Review Options. Final Report submitted to the Department of the Environment and Energy. 37 (Australia, 2017).

44 Anthony, K. R. N. Coral Reefs Under Climate Change and Ocean Acidification: Challenges and Opportunities for Management and Policy. *Annual Review of Environment and Resources* **41**, 59-81, doi:10.1146/annurev-environ-110615-085610 (2016).

45 Waterhouse, J. *et al.* 2017 Scientific Consensus Statement: Land use impacts on Great Barrier Reef water quality and ecosystem condition. Summary. 18 (Reef Water Quality Protection Plan Secretariat, Brisbane, Australia, 2017).

46 Brodie, J. *et al.* 2013 Scientific Consensus Statement: Land use impacts on Great Barrier Reef water quality and ecosystem condition. (Reef Water Quality Protection Plan Secretariat, The State of Queensland, Brisbane, Australia, 2013).

47 Brodie, J. E. *et al.* Terrestrial pollutant runoff to the Great Barrier Reef: An update of issues, priorities and management responses. *Marine Pollution Bulletin* **65**, 81-100, doi:10.1016/j.marpolbul.2011.12.012 (2012).

48 The State of Queensland and Commonwealth of Australia. 47 (Queensland Department of Premier and Cabinet, Brisbane, Australia, 2003).

49 Kroon, F. J., Thorburn, P., Schaffelke, B. & Whitten, S. Towards protecting the Great Barrier Reef from land-based pollution. *Global Change Biology* **22**, 1985-2002, doi:10.1111/gcb.13262 (2016).

50 Brodie, J. & Pearson, R. G. Ecosystem health of the Great Barrier Reef: Time for effective management action based on evidence. *Estuar. Coast. Shelf Sci.* **183**, 438-451, doi:10.1016/j.ecss.2016.05.008 (2016).

51 The Great Barrier Reef Water Science Taskforce. Great Barrier Reef Water Science Taskforce, Final Report, Clean water for a healthy reef. 94 (The Office of the Great Barrier Reef Department of Environment and Heritage Protection, Brisbane, Australia, 2016).

52 Reef Water Quality Protection Plan Secretariat. 2 (Reef Water Quality Protection Plan Secretariat, Brisbane, Australia, 2011).

53 Reef Water Quality Protection Plan Secretariat. 6 (Reef Water Quality Protection Plan Secretariat, Brisbane, Australia, 2013).

54 Reef Water Quality Protection Plan Secretariat. 6 (Reef Water Quality Protection Plan Secretariat, Brisbane, Australia, 2014).

55 Reef Water Quality Protection Plan Secretariat. Great Barrier Reef Report Card 2015. Reef Water Quality Protection Plan. 4 (Reef Water Quality Protection Plan Secretariat, Brisbane, Australia, 2016).

56 Office of the Great Barrier Reef. Results Great Barrier Reef Report Card 2016. Reef Water Quality Protection Plan. 155 (2017).

57 Queensland Government. Great Barrier Reef Report Card 2014. Reef Water Quality Protection Plan., 8 (Queensland Government, Brisbane, Australia, 2015).

58 Office of the Great Barrier Reef. Results Reef Water Quality Report Card 2017 and 2018. Reef 2050 Water Quality Improvement Plan. 413 (2019).

59 Gruber, R. *et al.* Marine Monitoring Program: Annual Report for inshore water quality monitoring 2017-18. Report for the Great Barrier Reef Marine Park Authority., 294 (Great Barrier Reef Marine Park Authority, Townsville, Australia, 2019).

60 Thompson, A., Costello, P., Davidson, J., Logan, M. & Coleman, G. Marine Monitoring Program. Annual Report for inshore coral reef monitoring: 2017 to 2018. 132 (Australian Institute of Marine Science, Townsville, 2019).
